# Phenotypical Characterization and Clinical Outcome of Canine Burkitt-Like Lymphoma

**DOI:** 10.3389/fvets.2021.647009

**Published:** 2021-03-17

**Authors:** Luca Aresu, Chiara Agnoli, Arturo Nicoletti, Antonella Fanelli, Valeria Martini, Francesco Bertoni, Laura Marconato

**Affiliations:** ^1^Department of Veterinary Sciences, University of Turin, Grugliasco, Italy; ^2^Department of Medical Veterinary Science, University of Bologna, Bologna, Italy; ^3^Department of Veterinary Medicine, University of Milan, Lodi, Italy; ^4^Institute of Oncology Research, Faculty of Biomedical Sciences, University of Italian Switzerland (USI), Bellinzona, Switzerland; ^5^Oncology Institute of Southern Switzerland, Bellinzona, Switzerland

**Keywords:** apoptic index, Burkitt-like lymphoma, caspase - 3, dog, MYC, prognosis

## Abstract

In dogs, Burkitt-like lymphoma (B-LL) is rare tumor and it is classified as a high-grade B-cell malignancy. The diagnosis is challenging because of the similar histologic appearance with other histotypes, no defined phenotypical criteria and poorly described clinical aspects. The aim of the study was to provide a detailed description of clinical and morphological features, as well as immunophenotypical profile of B-LL in comparison with the human counterpart. Thirteen dogs with histologically proven B-LL, for which a complete staging and follow-up were available, were retrospectively selected. Immunohistochemical expression of CD20, PAX5, CD3, CD10, BCL2, BCL6, MYC, and caspase-3 was evaluated. Histologically, all B-LLs showed a diffuse architecture with medium to large-sized cells, high mitotic rate and diffuse starry sky appearance. B-phenotype of neoplastic cells was confirmed both by flow-cytometry and immunohistochemistry. Conversely, B-LLs were negative for BCL2 and MYC, whereas some cases co-expressed BCL6 and CD10, suggesting a germinal center B-cell origin. Disease stage was advanced in the majority of cases. All dogs received CHOP-based chemotherapy with or without immunotherapy. Despite treatment, prognosis was poor, with a median time to progression and survival of 130 and 228 days, respectively. Nevertheless, ~30% of dogs survived more than 1 year. An increased apoptotic index, a high turnover index and caspase-3 index correlated with shorter survival. In conclusion, canine B-LL shows phenotypical differences with the human counterpart along with features that might help to differentiate this entity from diffuse large B-cell lymphoma.

## Introduction

In humans, Burkitt's lymphoma (BL) is classified as a highly aggressive B-cell lymphoma and was first described by Denis Burkitt in 1958. BL derives from mature germinal or post germinal center B-cells and is further divided into three clinical entities (sporadic, endemic, and immunodeficiency-associated), all having in common rearrangements of the *MYC* oncogene. Sporadic BL occurs in children and young adults, accounting for 30–50% of all lymphomas in children. It is more common in males than females. Endemic BL is typical of equatorial Africa and Papua New Guinea, with a higher incidence in children (4–7 years). Immunodeficiency-associated BL is more common in patients infected with human immunodeficiency virus (HIV), although its occurrence is not related to a low percentage of CD4 positive cells. The variants differ in their association with the Epstein Barr virus, which is present almost always in endemic BL tumor cells and in 20–40% of the other cases.

BL often presents as a rapidly growing tumor, being characterized by a very short doubling time and a quick dissemination to extranodal sites, including central nervous system (CNS) and bone marrow (BM) (1–4). Based on their origin, BL cells express PAX5, CD19, CD20, CD22, CD79a antigens, as well as germinal center origin antigens (CD10 and BCL6) (3, 5, 6). Proliferation rate (Ki-67-index) as well as apoptosis are markedly high. Morphologically, human BL is characterized by a diffuse architecture and severe tingible body starry sky appearance; furthermore, cells are monotonous with nuclei of intermediate size and a moderate volume of cytoplasm containing small lipid vacuoles. However, a proportion of cases may show variation in cellular, nuclear and nucleolar size, and some may have more prominent plasmacytoid features. These borderline cases, exhibiting some of the morphologic features of BL but lacking some of the characteristic immunophenotypic findings of classic BL, lie within a continuum between BL and diffuse large B-cell lymphoma (DLBCL), and are currently referred to unclassifiable aggressive B-cell lymphomas (7–9). These distinctions have significant therapeutic implications. Indeed, increasing knowledge of molecular drivers of lymphomas has allowed subclassification and opportunity for clinical investigations to personalize therapy in human patients and moving the field significantly forward (10). Unfortunately, despite improved delineation of lymphoid malignancy biology in dogs and the cooperative efforts of pathologists and oncologists worldwide, a long-standing problem refers to the description of specific canine entities, which occur rarely and for which the literature is scarce, including BL (11). Canine BL is described as a rare high-grade B-cell malignancy accounting for 2% of nodal canine lymphomas in dogs (12), but there is a very limited knowledge about its clinical behavior and response to therapy as well as its pathological and immunophenotypical features. Thus, the term Burkitt-like lymphoma (B-LL) is generally preferred to diagnose those borderline lymphomas of intermediate size, B-cell phenotype, and high mitotic rate that are not classified as DLBCL. In the World Health Organization (WHO) classification, canine B-LL was used to designate high-grade B-cell neoplasms that had the cellular size of classical BL but a variable nuclear size (13). Histologically, B-LL shows a diffuse architecture, a high mitotic rate and a starry sky pattern. Rarely, some centroblasts are present. The most consistent characteristic is anisokaryosis; nuclei show smooth contours without indentations. In contrast, DLBCL is characterized by the proliferation of medium to large B-cells consisting of centroblasts and immunoblasts; also, the starry sky appearance is less frequent. Furthermore, the mitotic count is quite heterogeneous compared with B-LL. These elements are generally taken into consideration when differentiating between the two histotypes (13). Due to the rarity of canine B-LL and the absence of dedicated studies, a detailed morphological description associated with immunophenotypic characterization is lacking. Also, prognostic information as well as therapeutic guidelines is currently unavailable to veterinary oncologists. To the authors' knowledge, only four studies have described canine B-LL or BL. In 2004, Ponce et al. (14) described 4 BLs, and in 2010 the same group of researchers expanded the BL population to 10 additional cases (15). In their series, BLs showed a marked starry-sky appearance and were composed by medium-sized cells with a diffuse infiltrative pattern, round nuclei, markedly clumped chromatin, multiple nucleoli, and a small ring of hyperbasophilic cytoplasm. Mitotic index was high, as was Ki-67 index. Extranodal involvement was common. In both studies, staging was not complete or treatment and outcome were not investigated. In 2013, Valli et al. (12) reported three B-LLs, which were grouped among high-grade lymphomas. Because of the above, no detailed information regarding staging and treatment response could be retrieved. Finally, one retrospective study including only BLs described a good response to chemotherapy, with 26% of dogs alive at more than 2 years (16). The results of this study were presented at a congress, and no follow-up study has been published. The aims of this retrospective study were to characterize the morphology and immunophenotypic profile in relation to the human counterpart and describe the clinical features and outcome variables, including time to progression (TTP) and lymphoma specific survival (LSS) of 13 dogs with B-LL receiving treatment.

## Materials and Methods

### Sources of Cases

Medical records were reviewed to identify dogs with treatment-naïve and histologically confirmed B-LL. All specimens were obtained under the formal consent from the owners. To be included in the analysis, dogs had to undergo a complete staging and lymphadenectomy of a peripheral enlarged lymph node (LN) with a final diagnosis of B-LL based on the WHO classification of canine lymphoma (17). Haematoxylin-eosin glass slides were reviewed by two independent pathologists (L.A., A.N.); discrepancies among authors were solved by consensus. Information on clinical stage was obtained by means of the following: hematological and biochemical analysis, thoracic radiographs, abdominal ultrasound, fine-needle aspiration of spleen and liver regardless of their sonographic appearance, fine needle aspiration of any altered organ, and BM evaluation.

### Flow Cytometry

Flow cytometry (FC) was performed on lymph node (LN) aspirates, BM and peripheral blood (PB) samples. Sample processing was performed with a multicolour approach, as previously described (18). Antibody clones, sources, and reactivity are listed in Supplementary Table 1. The extent of PB and BM infiltration was calculated as the percentage of cells showing the morphological and phenotypic characteristics detected in LN, out of the total CD45+ cells (19).

### Histologic Analysis

Histologic variables included mitotic count (MC) and apoptotic index (AI). MC was calculated as the absolute number of mitotic figures counted in 2.37 mm^2^ area (20). AI was calculated as the percentage of apoptotic cells and apoptotic bodies on the total number of tumor cells in in 2.37 mm^2^ area; areas of highest apoptotic activity were selected (21).

### Immunohistochemistry

For immunohistochemistry (IHC), serial paraffin sections were processed as previously described (22) using an automatic immunostainer (Ventana Benchmark XT, Ventana Medical Systems Inc.). Immunohistochemical analysis was performed in accordance with the guidelines of the American Association of Veterinary Diagnosticians (AAVLD) Subcommittee on Standardization of Immunohistochemistry (23). The following markers were assessed by IHC: CD3, CD10, CD20, BCL2, BCL6, PAX5, MUM1, Ki-67, caspase-3 and MYC. The panel of antibodies was also employed in a reactive LN to define the IHC profile. Supplementary Table 2 lists sources, clones, dilution for each antibody and references in the canine species. IHC-labeled slides were blindly reviewed by three authors (L.A., A.N., A.F.). The immunoreactivity for CD20, CD10, BCL2, BCL6, PAX5, MUM1, and MYC was assessed as positive if >50% of neoplastic cells showed strong, diffuse, and marker-specific labeling. The Ki-67 index was determined by counting the number of positive cells per 1,000 randomly selected cells excluding necrotic areas. The caspase-3 and CD3 index was calculated as the percentage of positive cells in 10 consecutive high-power fields (x40); the most intense stained areas were selected. Finally, the percentage of Ki-67 positive cells and AI were coupled to calculate proliferation/apoptosis ratio (PAR, % Ki-67 positive cells/AI) and turnover index (TI, % Ki-67 positive cells plus AI) (24).

### Treatment

All dogs were treated with chemotherapy with or without immunotherapy (25) and had a complete follow-up. Treatment response was classified as complete remission (CR), partial remission (PR), stable disease (SD) or progressive disease (PD) (26). Response was evaluated at each therapeutic session and was required to last for at least 28 days. Follow-up evaluation consisted of monthly physical examination, peripheral LNs size measurement and cytological evaluation during the first year, and every other month thereafter. Relapse was defined as the clinical reappearance and cytological evidence of lymphoma with or without FC confirmation in any anatomical site in dogs having experienced complete remission (CR). Once relapse was confirmed, a complete restaging work-up was undertaken, and a second round of chemotherapy was offered.

### Statistical Analysis

TTP was calculated as the interval between initiation of treatment and PD or relapse, whereas LSS was measured as the interval between initiation of treatment and lymphoma-related death. Dogs lost to follow-up or dead for lymphoma-unrelated causes before PD, as well as those still in CR at the end of the study, were censored for TTP analysis. Dogs alive at the end of the study, lost to follow-up or dead due to causes other than lymphoma were censored for LSS analysis.

Response rate (RR) was defined as the sum of all dogs achieving CR and PR. Survival was analyzed according to the method of Kaplan-Meier. Univariate Cox's proportional hazard regression analysis was performed to determine possible associations between TTP or LSS and the following variables: MC (absolute value), AI (%), Ki67 index (%), PAR, TI, caspase-3 index, BCL6 expression (positive or negative), CD3-positive cells (%). Significance was set at *P* < 0.05. A Shapiro-Wilk test was performed to assess normal distribution of AI and caspase-3 index. Thereafter, Pearson's test was performed to assess the degree of linear correlation between them. All statistical analysis was performed using SPSS Software version 20.0.

## Results

### Clinical Results

Between 2012 and 2019, 13 dogs with B-LL were identified. There were three (23.1%) mixed-breed dogs, three (23.1%) Golden retrievers, and one (7.7%) each of the following: Beagle, Dobermann, Basset Hound, Rottweiler, Labrador retriever, Welsh Corgi, and Galgo. Seven (53.8%) dogs were male (one castrated), and six (46.2%) were female (five spayed). Median age was 9 years (range, 5–12 years) and median weight was 30.8 kg (range, 9.1–41.8 kg). At admission, all dogs had generalized peripheral lymphadenopathy of 7–90 days duration (median, 20 days). Eight (61.5%) dogs were asymptomatic (substage a), whereas five (38.5%) dogs showed clinical signs (substage b). Anemia (PCV <35%) was documented in two (15.4%) dogs, thrombocytopenia in three (23.1%), and increased LDH level in six (46.2%). None of the dogs was hypercalcaemic. The majority of cases had stage V (*n* = 8, 61.5%) or IV (*n* = 4, 30.8%) disease, with only one dog (7.7%) having stage III disease. Three (23.1%) dogs had pulmonary infiltration at diagnosis. Signalment, clinical presentation, clinico-pathological variables, and outcome for each case are listed in Supplementary Table 3.

### Cytopathology and Flow Cytometry

**Figure 1:**
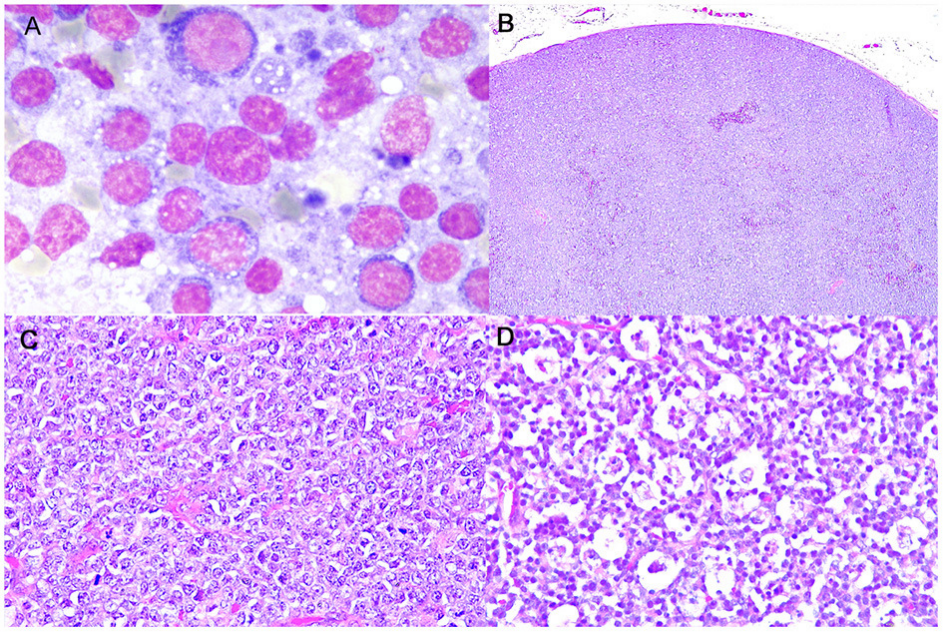
Burkitt-like lymphoma (B-LL), lymph node, dog. **(A)** Medium-sized cells, with round nuclei, scant deeply basophilic cytoplasm and mitotic figure. MayGrunwald-Giemsa, x100 magnification. **(B)** Sheets of densely packed neoplastic cells effacing the entire lymph node and compressing the peripheral sinus. Haematoxylin and eosin (HE), x4 magnification. **(C)** Intermediate to large neoplastic lymphoid cells with distinct cell borders, moderate anisokaryosis with cleaved or oval-shaped nuclei and vesicular appearance. HE, x40 magnification. **(D)** Numerous tingible body macrophages phagocytizing apoptotic debris and starry-sky appearance. HE, x40 magnification.

Cytologically, lymphomas were characterized by a prevalent population of medium-sized cells, having round nuclei with clumped chromatin and a scant, deeply basophilic, occasionally vacuolated cytoplasm. Nucleoli were inconsistently observed. Mitoses were frequently identified (Figure 1A).

FC on LN aspirates revealed a dominant population (>60%) of cells being larger in size than residual T-lymphocytes. These cells were consistently positive for CD45, negative for CD5 and CD34, and showed a higher expression of CD21 compared with residual small-sized B-cells. Two B-LLs were also tested for Ki-67 expression (27), revealing a high-grade lymphoma (27.0 and 29.7%, respectively).

Median PB infiltration was 1.4% (range, 0.2–17.6%), whereas median BM infiltration was 0.9% (range, 0.2–35.4%). Seven (53.8%) PB samples and five (38.5%) BM samples had a percentage of large CD21-positive cells exceeding those encountered in non-neoplastic controls (28), thus being considered positive for infiltration.

### Histopathology

Histologically, LNs were characterized by a diffuse proliferation of intermediate to large-sized cells, with moderately abundant cytoplasm and frequent vesicular appearance round to oval nuclei, presenting multiple nucleoli and only rarely a single nucleolus (Figures 1B,C). Moderate anisokaryosis was observed and the MC ranged from 5 to 10 (mean 7, median 6). Necrosis occupied only small focal areas of the sections. Numerous tingible body macrophages, phagocytizing abundant apoptotic debris and creating a starry-sky pattern, were observed diffusely (Figure 1D) and AI ranged from 23.5 to 68.5% (mean 42.5%, median 39.5%). All examined histologic variables are shown in Supplementary Table 4.

### Immunohistochemistry

**Figure 2:**
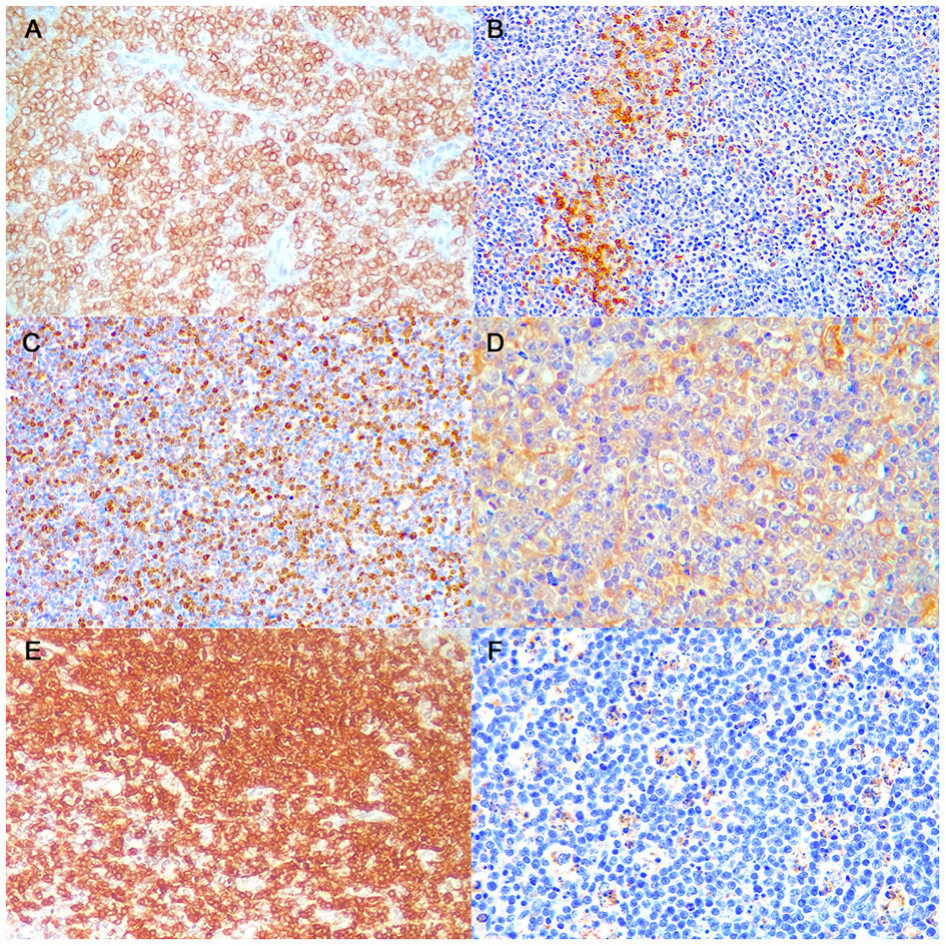
Burkitt-like lymphoma (B-LL), lymph node, dog. Representative images of CD20, CD3, Ki-67, CD10, BCL6, and caspase-3 immunoreactivity, x40 magnification. **(A)** The neoplastic cells show strong membranous positive immunolabeling. Immunohistochemistry (IHC) for CD20. **(B)** Few small lymphocytes show cytoplasmatic positive immunolabeling. IHC for CD3. **(C)** The neoplastic cells show multifocal to diffuse nuclear immunolabeling. IHC for Ki-67. **(D)** The neoplastic cells show diffuse cytoplasmic positive immunolabeling. IHC for CD10. **(E)** The neoplastic cells show diffuse cytoplasmic positive immunolabeling. IHC for BCL6. **(F)** Tingible body macrophages and apoptotic debris distinctly immunostained. IHC for caspase-3.

A detailed description of immunohistochemical results are shown in Table 1. All B-LLs stained strongly and diffusely with CD20 (Figure 2A) and PAX5; conversely, neoplastic cells were negative to CD3. Few small CD3 positive lymphocytes were always observed within the tumor (Figure 2B). The mean rate of CD3 index was 10% by IHC (range, 5–25%) and 7.5% by FC (range, 2.5–14.2%). Ki-67 index ranged from 17.1 to 42.7% (mean 32.8%, median 35.8) (Figure 2C). Mean PAR value was 0.9 (median 0.8; range, 0.4–1.7) and mean TI was 75.4% (median 69.1%; range 43.1–101.3%). CD10 staining was diffusely positive (Figure 2D), whereas only six cases showed a diffuse cytoplasmic labeling for BCL6 (Figure 2E). Conversely, all B-LLs were negative for MUM1, BCL2, and MYC. Finally, caspase-3 index was evaluated similarly to AI and a linear correlation was identified *(P* = 0.009, *r*^2^ = 0.513) (Figure 2F).

**Table 1:** Immunohistochemical profile of 13 canine Burkitt-like lymphomas.

**No**.	**CD1^†^**	**CD20^†^**	**PAX5^†^**	**BCL2^†^**	**BCL6^†^**	**MUM1^†^**	**MYC^†^**	**CD3 (%)^‡^**	**Ki-67 (%)^§^**	**caspase-3 (%)^‡^**
1	+	+	+	–	+	–	–	15	25.6	n/d
2	+	+	+	–	–	–	–	10	36.3	37.5
3	+	+	+	–	+	–	–	5	36.3	48.5
4	+	+	+	–	+	–	–	5	31.6	36.5
5	+	+	+	–	+	–	–	20	21.6	45
6	+	+	+	–	–	–	–	10	43.8	54
7	+	+	+	–	+	–	–	25	33.1	48
8	+	+	+	–	+	–	–	5	34.8	55
9	n/d	+	+	–	–	–	–	5	42.1	28.5
10	n/d	+	+	–	–	–	–	10	17.1	14
11	n/d	+	+	–	–	–	–	5	21.7	24
12	n/d	+	+	–	–	–	–	5	42.7	40.5
13	+	+	+	–	–	–	–	10	40.3	27.5

† *Scoring: –, <50% positive cells; +, >50% positive cells*.

‡ *Percentage of positive cells per 10 consecutive high-power fields (x40)*.

§ *Percentage of positive cells per 1,000 randomly selected cells*.

### Treatment and Outcome

Two (15.4%) dogs had received steroids before admission. All dogs were treated with a CHOP-based protocol of various dose-intensity, consisting of an induction phase of 12–19 weeks with no maintenance phase. Five (38.5%) dogs also received a concomitant autologous vaccine with hydroxylapatite and tumor-derived heat shock protein-peptide complexes. The RR was 92.3% (12/13), consisting of 10 (76.9%) CR and two (15.4%) PR. Only one (7.7%) dog progressed. The median TTP was 130 days (range, 1–493 days) and median LSS was 228 days (range, 14–733 days). All dogs relapsed before data analysis closure. At the end of the study, 10 (76.9%) dogs had died for their lymphoma and three (23.1%) dogs were still alive after 446, 537, and 733 days, respectively. Among those, two received concurrent immunotherapy.

Among the tested variables, increased AI correlated with shorter TTP *(P* = 0.016, HR 1.058, 95% CI 1.011–1.108) and LSS *(P* = 0.045, HR 1.042, 95% CI 1.001–1.085), and a high TI value correlated with shorter LSS *(P* = 0.041, HR 1.040, 95% CI 1.002–1.080). Moreover, increasing of caspase-3 index was associated with shorter LSS *(P* = 0.038, HR 1.072, 95% CI 1.004–1.146).

## Discussion

In 2008, a high level of reproducibility of canine lymphoma diagnosis fulfilling the morphological criteria described in the WHO classification was reported (29). While for the six most common diagnoses, the overall agreement level reached 87% (29), B-LL and BL were not specifically discussed and were considered a together with DLBCL. Here we report the clinical, histological and immunohistochemical characterization of a canine subset of MYC and BCL2-negative, high-grade B-cell lymphomas sharing similarities with B-LL.

Applying the WHO morphological criteria, all lymphomas showed a diffuse growth pattern. When compared to the study of Valli et al. (12), MC was moderately lower, however the counting method in our case series was different, since we evaluated a defined area of 2.37 mm^2^ for the analysis. Also, analogous comparison cannot be performed with Ponce et al. (15) data because the proliferation rate was expressed as mitotic index. Similarly to MC, Ki-67 index was severely affected by the presence of macrophages and, when recalculating the index excluding these cells, the index increased to 84%. However, an association between Ki-67 index and outcome was not identified. Conversely, an increased AI correlated with shorter TTP and LSS. To date, in human oncology it is still controversial whether a relationship exists between apoptosis and prognosis, but, as a general rule, an increased AI is associated with a worse clinical outcome (30) and this may hold true in canine B-LL as well. In addition, a correlation between AI and caspase-3 index was observed in the current series. Caspase-3 immunoreactivity was identified in the cytoplasm of apoptotic cells and occasionally neoplastic lymphocytes showed nuclear staining. Immunoreactivity was also evident in a few cells having a normal cellular and nuclear morphology, reflecting an early stage of apoptosis. Interestingly, TI was associated with LSS, possibly predicting the clinical response in dogs undergoing treatment. However, a larger number of cases is needed to reinforce these data.

In recent years, several groups have attempted to discover molecular similarities of the most frequent canine B-cell lymphoma histotypes with the human counterparts, but B-LL was never considered (31, 32). This is attributable to the low incidence of this particular lymphoma subtype and the diagnostic conundrum to frame such lesion. Here, we investigated the immunophenotypic profile and included a panel of antibodies that are routinely used to characterize human BL. In details, BCL6 and CD10 positive expression associated with BCL2 negative immunostaining are considered highly suggestive of BL in people. BCL2 protein was absent in all samples of the present study. Conversely, Curran et al. (33) reported a large number of canine DLBCL expressing BCL2. Even if a larger number of cases is needed to confirm this result, BCL2 immunostaining might represent a distinctive feature for differentiating these two histotypes in dogs. BCL2 is typically not expressed in human BL, but a weak positivity is still considered compatible with this diagnosis if clinical, morphological and further immunohistochemical characteristics are consistent with it. Conversely, if BCL2 intensity is moderate to strong and Ki-67 is <95%, a diagnosis of unclassifiable B-cell lymphoma is given with features in between DLBCL and BL (3). The immunostaining of BCL6 was heterogeneous in our cases, whereas CD10 was always expressed. At least, for the BCL6 and CD10 double positive B-LLs, an origin from germinal center B-cells may be hypothesized. However, more advanced technology, such as RNA-seq, is needed to accurately determine the cell of origin.

MYC protein was not expressed in our case series, and indeed there are no reports in the literature of the canine homolog of the human *t*(8;14)(q24;q32) chromosomal translocation juxtaposing the *MYC* oncogene to the immunoglobulin heavy chain genes locus. While MYC is highly expressed in canine DLBCL (31), its lack of expression in B-LL is striking. However, this peculiar behavior was previously observed both in human HIV-B-LL where *MYC* genetic lesions were not identified and in Simian negative immunodeficiency virus-infected rhesus monkeys B-LL (34, 35).

In this respect, other members of the *MYC* family, *MYCN* and *MYCL*, have similar oncogenic potential (36–38) and could be the drivers of the canine disease compensating the absence of MYC protein, sharing targets and activating similar pathways. Although *MYCN* is more commonly deregulated in human solid tumors (especially for childhood onset) (36), it has also been reported translocated in human lymphomas (39, 40), mouse plasma cell tumors (41, 42), and, importantly, expressed in the very few human BL cases apparently lacking *MYC* deregulation (43, 44). Future studies are needed to define this peculiar observation of MYC negativity in canine B-LL.

The B-cell phenotype of B-LL was always confirmed by FC and IHC. The staining for CD20 and PAX5 was diffuse and intense in neoplastic cells. Few CD3+ T cells infiltrating the LN were always retrieved, but differently from DLBCL, no association with prognosis was identified.

From a clinical standpoint, approximately 40% of dogs showed symptoms and had widespread disease at admission, indicating the aggressive nature of canine B-LL. Three (23%) dogs had pulmonary involvement; this finding is in line with the human counterpart, where 16% of patients have pleural/pulmonary involvement (45). In the current series, seven (53.8%) and five (38.5%) dogs had PB and BM involvement, respectively, similarly to what has been reported for canine DLBCL (46).

Given the lack of studies specifically focusing on B-LL, there is currently no gold standard treatment. All dogs in our study were treated with a CHOP-based chemotherapy, consisting of cyclophosphamide, vincristine, doxorubicin and prednisone, and five of them concurrently received an HSPPC autologous vaccine with hydroxylapatite and tumor-derived heat shock protein-peptide complexes. Despite treatment, the prognosis was disappointingly poor, with a median TTP and median LSS of 130 and 228 days, respectively. Nevertheless, three (23.1%) dogs survived more than 1 year, and one (7.7%) dog was alive after 2 years from diagnosis, suggesting that a long survival may be achieved in some dogs despite relapse. Of note, these four dogs had stage V disease and three of them received chemo-immunotherapy; further studies are warranted to confirm the role of active immunotherapy for the treatment of B-LL.

In the only published study in which outcome has been described (14), the median OS was 15 days, and the dog that lived longer survived for 21 days only. Compared with the dogs of our series, the outcome was significantly worse. There are several reasons behind these findings. In that previous study, all dogs were severely symptomatic and had intestinal involvement; clinical signs had been present for <1 week. In our series, the majority of dogs were asymptomatic, and none had alimentary involvement. Second, doxorubicin was scheduled to be introduced only after the second relapse, which means that none of the dogs had received one of the most effective drugs against lymphoma, which may have accounted for the worse prognosis. Another explanation for discrepant outcomes between previous and current datasets relies on MYC expression. As outlined in human studies, MYC-positive BLs have a worse outcome compared with MYC-negative BLs (47). While in the Ponce study MYC expression was not investigated, all dogs in the current series were MYC-negative, possibly having a better treatment response.

In human patients with BL, central nervous system (CNS) prophylaxis with intrathecal methotrexate or cytarabine is a component of first-line CHOP chemotherapy, given the high risk of CNS involvement or relapse (6, 48, 49). In the current series, although autopsy or cerebrospinal fluid analysis was not carried out in any of the dogs, CNS involvement was not clinically evident.

Finally, unlike human BL, results from previous canine studies tend to exclude an association between Epstein Barr virus (EBV) and lymphoma. This was demonstrated in dogs both by cancer virome analysis and investigating specifically EBV in lymphoma samples (50).

## Conclusions

Herein we report the clinical and pathological features of 13 canine B-LLs. Similarities with human BL were observed both at histological and immunophenotypical level, but one main difference was the absence of MYC expression. Caution is warranted before concluding that canine B-LL indeed represent true MYC-negative BL. The cases described in the current series may rather represent a distinct subset of MYC-negative unclassifiable high-grade B-cell lymphomas. The high AI and caspase-3 index should be considered as a valid feature to differentiate canine B-LL from DLBCL in association with the lack of BCL2 expression. Given these preliminary results, we suggest using the term of B-LL over BL until a more accurate molecular and genetic description of this lymphoma histotype is available. Finally, due to the rarity of canine B-LL, a cooperative effort among researcher groups worldwide is critical if we are to move the bar higher for meaningfully treating each subtype of aggressive lymphoma.

## Data Availability Statement

The raw data supporting the conclusions of this article will be made available by the authors, without undue reservation.

## Author Contributions

LA, FB, and LM: conceptualization. CA, VM, AN, and AF: methodology. VM, AN, and AF: analysis. LA, FB, and LM: writing, review, and editing. All authors contributed to the article and approved the submitted version.

## Conflict of Interest

The authors declare that the research was conducted in the absence of any commercial or financial relationships that could be construed as a potential conflict of interest.

## References

[1] Mbulaiteye SM Anderson WF Ferlay J Bhatia K Chang C Rosenberg PS Devesa SS Parkin DM. Pediatric, elderly, and emerging adult-onset peaks in Burkitt's lymphoma incidence diagnosed in four continents, excluding Africa. Am J Hematol. (2012) 87:573–8. 10.1002/ajh.23187 22488262 PMC3358448

[2] Ogwang MD Bhatia K Biggar RJ Mbulaiteye SM. Incidence and geographic distribution of endemic Burkitt lymphoma in northern Uganda revisited. Int J Cancer. (2008) 123:2658–63. 10.1002/ijc.23800 18767045 PMC2574984

[3] Leoncini L Campo E Stein H Harris NL Jaffe ES Kluin PM. WHO Classification of Tumours of Haematopoietic and Lymphoid Tissues. In: Swerdlow SH Campo E Nancy LH editors. Burkitt's Lymphoma. WHO (2017). p. 330–334.

[4] Brady G MacArthur GJ Farrell PJ. Epstein-Barr virus and Burkitt lymphoma. Postgrad Med J. (2008) 84:372–7. 10.1136/jcp.2007.047977 18716017

[5] Hecht JL Aster JC. Molecular biology of Burkitt's Lymphoma. J Clin Oncol. (2000) 18:3707–21. 10.1200/JCO.2000.18.21.3707 11054444

[6] Bernstein JI Coleman CN Strickler JG Dorfman RF Rosenberg SA. Combined modality therapy for adults with small noncleaved cell lymphoma (Burkitt's and non-Burkitt's types). J Clin Oncol. (1986) 4:847–58. 10.1200/JCO.1986.4.6.847 3711961

[7] Jaffe ES. The 2008 WHO classification of lymphomas: implications for clinical practice and translational research. Hematology Am Soc Hematol Educ Program. (2009) 2009:523–31. 10.1182/asheducation-2009.1.523 20008237 PMC6324557

[8] Braziel RM Arber DA Slovak ML Gulley ML Spier C Kjeldsberg C. The Burkitt-like lymphomas: a Southwest Oncology Group study delineating phenotypic, genotypic, and clinical features. Blood. (2001) 97:3713–20. 10.1182/blood.V97.12.3713 11389007

[9] Haralambieva E Boerma E-J van Imhoff GW Rosati S Schuuring E Müller-Hermelink HK. Clinical, immunophenotypic, and genetic analysis of adult lymphomas with morphologic features of Burkitt lymphoma. Am J Surg Pathol. (2005) 29:1086–1094. 10.1097/01.pas.0000168176.71405.e5 16006805

[10] LaCasce AS Kho ME Friedberg JW Niland JC Abel GA Rodriguez MA. Comparison of referring and final pathology for patients with non-Hodgkin's lymphoma in the national comprehensive cancer network. J Clin Oncol. (2008) 26:5107–12. 10.1200/JCO.2008.16.4061 18768434 PMC2652094

[11] Comazzi S Marconato L Argyle DJ Aresu L Stirn M Grant IA. The European canine lymphoma network: a joining initiative to generate consensus guidelines for the diagnosis and therapy in canine lymphoma and research partnership. Vet Comp Oncol. (2015) 13:494–7. 10.1111/vco.12128 26463403

[12] Valli VE Kass PH Myint MS Scott F. Canine lymphomas: association of classification type, disease stage, tumor subtype, mitotic rate, and treatment with survival. Vet Pathol. (2013) 50:738–48. 10.1177/0300985813478210 23444036

[13] Valli VE editor. Veterinary Comparative Hematopathology. Oxford: Blackwell Publishing Ltd. (2007). 10.1002/9780470344545

[14] Ponce F Magnol JP Ledieu D Marchal T Turinelli V Chalvet-Monfray K. Prognostic significance of morphological subtypes in canine malignant lymphomas during chemotherapy. Vet J. (2004) 167:158–66. 10.1016/j.tvjl.2003.10.009 14975390

[15] Ponce F Marchal T Magnol JP Turinelli V Ledieu D Bonnefont C. A morphological study of 608 cases of canine malignant lymphoma in France with a focus on comparative similarities between canine and human lymphoma morphology. Vet Pathol. (2010) 47:414–33. 10.1177/0300985810363902 20472804

[16] Hershey A Valli V. Prognosis of canine burkitt-like lymphoma treated with CHOP chemotherapy. In: Veterinary Cancer Society Conference. Paris (2012).

[17] Valli VE Myint M Barthel A Bienzle D Caswell J Colbatzky F. Classification of canine malignant lymphomas according to the world health organization criteria. Vet Pathol. (2011) 48:198–211. 10.1177/0300985810379428 20861499

[18] Marconato L Comazzi S Aresu L Riondato F Stefanello D Ferrari R. Prognostic significance of peripheral blood and bone marrow infiltration in newly-diagnosed canine nodal marginal zone lymphoma. Vet J. (2019) 246:78–84. 10.1016/j.tvjl.2019.02.002 30902194

[19] Cozzi M Marconato L Martini V Aresu L Riondato F Rossi F. Canine nodal marginal zone lymphoma: descriptive insight into the biological behaviour. Vet Comp Oncol. (2018) 16:246–52. 10.1111/vco.12374 29205839

[20] Meuten DJ Moore FM George JW. Mitotic count and the field of view area: time to standardize. Vet Pathol. (2016) 53:7–9. 10.1177/0300985815593349 26712813

[21] Archana M Bastian, Yogesh TL Kumaraswamy KL. Various methods available for detection of apoptotic cells-a review. Indian J Cancer. (2013) 50:274–83. 10.4103/0019-509X.118720 24061471

[22] Aresu L Martini V Rossi F Vignoli M Sampaolo M Aricò A. Canine indolent and aggressive lymphoma: clinical spectrum with histologic correlation. Vet Comp Oncol. (2015) 13:348–62. 10.1111/vco.12048 23782432

[23] Ramos-Vara JA Kiupel M Baszier T Bliven L Brodersen B Chelack B. Suggested guidelines for immunohistochemical techniques in veterinary diagnostic laboratories. J Vet Diagnostic Investig. (2008) 20:393–413. 10.1177/104063870802000401 18599844

[24] Phillips BS Kass PH Naydan DK Winthrop MD Griffey SM Madewell BR. Apoptotic and proliferation indexes in canine lymphoma. J Vet Diagnostic Investig. (2000) 12:111–7. 10.1177/104063870001200202 10730938

[25] Marconato L Frayssinet P Rouquet N Comazzi S Leone VF Laganga P. Randomized, placebo-controlled, double-blinded chemoimmunotherapy clinical trial in a pet dog model of diffuse large B-cell lymphoma. Clin Cancer Res. (2014) 20:668–77. 10.1158/1078-0432.CCR-13-2283 24300788

[26] Vail DM Michels GM Khanna C Selting KA London CA. Response evaluation criteria for peripheral nodal lymphoma in dogs (v1.0)-a veterinary cooperative oncology group (VCOG) consensus document. Vet Comp Oncol. (2010) 8:28–37. 10.1111/j.1476-5829.2009.00200.x 20230579

[27] Poggi A Miniscalco B Morello E Comazzi S Gelain ME Aresu L. Flow cytometric evaluation of ki67 for the determination of malignancy grade in canine lymphoma. Vet Comp Oncol. (2015) 13:475–80. 10.1111/vco.12078 24341365

[28] Riondato F Miniscalco B Poggi A Aricò A Aresu L Comazzi S. Analytical and diagnostic validation of a flow cytometric strategy to quantify blood and marrow infiltration in dogs with large B-Cell lymphoma. Cytom Part B - Clin Cytom. (2016) 90:525–30. 10.1002/cyto.b.21353 26663674

[29] Valli V. Veterinary pathologists achieve 80% agreement in application of WHO diagnoses to canine lymphoma. Cancer Ther. (2008) 6:221–6.

[30] Viswanathan V Juluri R Goel S Madan J Mitra SK Gopalakrishnan D. Apoptotic index and proliferative index in premalignant and malignant squamous cell lesions of the oral cavity. J Int oral Heal JIOH. (2015) 7:40–3. 25709366 PMC4336659

[31] Aresu L Ferraresso S Marconato L Cascione L Napoli S Gaudio E. New molecular and therapeutic insights into canine diffuse large B-cell lymphoma elucidates the role of the dog as a model for human disease. Haematologica. (2019) 104:e256–9. 10.3324/haematol.2018.207027 30545928 PMC6545862

[32] Giannuzzi D Giudice L Marconato L Ferraresso S Giugno R Bertoni F. Integrated analysis of transcriptome, methylome and copy number aberrations data of marginal zone lymphoma and follicular lymphoma in dog. Vet Comp Oncol. (2020) 18:645–55. 10.1111/vco.12588 32154977

[33] Curran KM Schaffer PA Frank CB Lana SE Hamil LE Burton JH. BCL2 and MYC are expressed at high levels in canine diffuse large B-cell lymphoma but are not predictive for outcome in dogs treated with CHOP chemotherapy. Vet Comp Oncol. (2017) 15:1269–79. 10.1111/vco.12263 27514648

[34] Kahnt K Mätz-Rensing K Hofmann P Stahl-Hennig C Kaup FJ. SIV-associated lymphomas in rhesus monkeys (Macaca mulatta) in comparison with HIV-associated lymphomas. Vet Pathol. (2002) 39:42–55. 10.1354/vp.39-1-42 12102218

[35] Carbone A Gaidano G Gloghini A Pastore C Saglio G Tirelli U. BCL-6 protein expression in AIDS-related non-Hodgkin's lymphomas: inverse relationship with Epstein-Barr virus-encoded latent membrane protein- 1 expression. Am J Pathol. (1997) 150:155–65. 9006332 PMC1858533

[36] Ruiz-Pérez MV Henley AB Arsenian-Henriksson M. The MYCN protein in health and disease. Genes (Basel). (2017) 8:1–27. 10.3390/genes8040113 PMC5406860 28358317

[37] Rickman DS Schulte JH Eilers M. The expanding world of N-MYC-driven tumors. Cancer Discov. (2018) 8:150–64. 10.1158/2159-8290.CD-17-0273 29358508

[38] Bachmann AS Geerts D. Polyamine synthesis as a target of MYC oncogenes. J Biol Chem. (2018) 293:18757–69. 10.1074/jbc.TM118.003336 30404920 PMC6290138

[39] Brown A Sciascia-Visani I Farrell D Smith M Felix C Mutharajah V. A patient with a diagnosis of nodal marginal zone B-cell lymphoma and a t(2;14)(p24;q32) involving MYCN and IGH. Mol Cytogenet. (2019) 12:12–6. 10.1186/s13039-019-0419-3 30733831 PMC6359751

[40] Wlodarska I Dierickx D Vanhentenrijk V Van Roosbroeck K Pospíšilová H Minnei F. Translocations targeting CCND2, CCND3, and MYCN do occur in t(11;14)-negative mantle cell lymphomas. Blood. (2008) 111:5683–90. 10.1182/blood-2007-10-118794 18391076

[41] Axelson H Wang Y Silva S Mattei M -G Klein G. Juxtaposition of N-myc and Igk through a reciprocal t(6;12) translocation in a mouse plasmacytoma. Genes Chromosom Cancer. (1994) 11:85–90. 10.1002/gcc.2870110204 7529553

[42] Kovalchuk AL DuBois W Mushinski E McNeil NE Hirt C Qi CF. AID-deficient Bcl-xL transgenic mice develop delayed atypical plasma cell tumors with unusual Ig/Myc chromosomal rearrangements. J Exp Med. (2007) 204:2989–3001. 10.1084/jem.20070882 17998390 PMC2118515

[43] Mundo L Ambrosio MR Raimondi F Del Porro L Guazzo R Mancini V. Molecular switch from MYC to MYCN expression in MYC protein negative Burkitt lymphoma cases. Blood Cancer J. (2019) 9:91. 10.1038/s41408-019-0252-2 31748534 PMC6868231

[44] De Falco G Ambrosio MR Fuligni F Onnis A Bellan C Rocca BJ. Burkitt lymphoma beyond MYC translocation: N-MYC and DNA methyltransferases dysregulation. BMC Cancer. (2015) 15:1–13. 10.1186/s12885-015-1661-7 26453442 PMC4600215

[45] Mian M Wasle I Gritsch S Willenbacher W Fiegl M. B cell lymphoma with lung involvement: what is it about? Acta Haematol. (2015) 133:221–5. 10.1159/000365778 25376208

[46] Marconato L Aresu L Stefanello D Comazzi S Martini V Ferrari R. Opportunities and challenges of active immunotherapy in dogs with B-cell lymphoma: a 5-year experience in two veterinary oncology centers. J Immunother Cancer. (2019) 7:1–9. 10.1186/s40425-019-0624-y 31174615 PMC6554898

[47] Salaverria I Martin-Guerrero I Wagener R Kreuz M Kohler CW Richter J. A recurrent 11q aberration pattern characterizes a subset of MYC-negative high-grade B-cell lymphomas resembling Burkitt lymphoma. Blood. (2014) 123:1187–98. 10.1182/blood-2013-06-507996 24398325 PMC3931189

[48] Hill QA Owen RG. CNS prophylaxis in lymphoma: who to target and what therapy to use. Blood Rev. (2006) 20:319–32. 10.1016/j.blre.2006.02.001 16884838

[49] Sariban E Edwards B Janus C Mograth I. Central nervous system involvement in American Burkitt's lymphoma. J Clin Oncol. (1983) 1:677–81. 10.1200/JCO.1983.1.11.677 6689423

[50] Milman G Smith KC Erles K. Serological detection of Epstein-Barr virus infection in dogs and cats. Vet Microbiol. (2011) 150:15–20. 10.1016/j.vetmic.2010.12.013 21242039

