# Supplementary material for: Phenotypical Characterization and Clinical Outcome of Canine Burkitt-Like Lymphoma
Source: Front Vet Sci. 2021 Mar 17;8:647009. doi: 10.3389/fvets.2021.647009 (PMC8010238; doi:10.3389/fvets.2021.647009)
Supplement: Supplementary file 1 [file Data_Sheet_1.docx]

**Table S1.** Antibody panel used for flow cytometric analysis of lymph node aspirates in 13 dogs with histopathologically proven Burkitt-like lymphoma.

| **Target** | **Antibody clone** | **Source** | **Reactivity** |
| --- | --- | --- | --- |
| CD21 | CA2.1D6 | Bio-Rad, Oxford, UK | Mature B-lymphocytes |
| CD5 | YKIX322.3 | Bio-rad | T-cells |
| CD45 | YKIX716.13 | Bio-rad | All leukocytes |
| MHC II | YKIX334.2 | Bio-rad | Monocytes, Lymphocytes |
| CD34 | 1H6 | BD Pharmingen, San Diego, CA, USA | Precursors |

**Table S2**. Antibody panel used for immunohistochemistry.

| **Antibody** | **Manufacturer** | **Source/Isotype** | **Clone** | **Antigen retrieval** | **Dilution** | **Reference** |
| --- | --- | --- | --- | --- | --- | --- |
| CD3 | Dako, Glostrup, Denmark | Mouse, IgG1 | F7.2.38 | Tris-EDTA buffer, pH 9.0 | 1:30 | Canine indolent and aggressive lymphoma: clinical spectrum with histologic correlation. Aresu L, et al. Vet Comp Oncol. 2015 Dec;13(4):348-62. |
| CD10 | Abclonal, Woburn, MA, USA | Rabbit, IgG | Polyclonal | Citrate buffer, pH 6.0 | 1:200 | Pax8, Napsin A, and CD10 as Immunohistochemical Markers of Canine Renal Cell Carcinoma. Peat TJ, et al. Vet Pathol. 2017 Jul;54(4):588-594 |
| CD20 | Thermo Fischer, Ashfor, UK | Rabbit, IgG | Polyclonal | Ventana Benchmark XT | 1:800 | Canine indolent and aggressive lymphoma: clinical spectrum with histologic correlation. Aresu L, et al. Vet Comp Oncol. 2015 Dec;13(4):348-62. |
| BCL2 | Sigma Aldrich, Suffolk, UK | Mouse, IgG1 | Monoclonal | Ventana Benchmark XT | 1:300 | Expression of Ki67, BCL-2, and COX-2 in canine cutaneous mast cell tumors: association with grading and prognosis. Vascellari M, et al. Vet Pathol. 2013 Jan;50(1):110-21. |
| BCL6 | Abcam, Cambridge, UK | Rabbit, IgG | Monoclonal | Ventana Benchmark XT | 1:100 | Evaluation of the prognostic significance of BCL6 gene expression in canine high-grade B-cell lymphoma. Sato M. et al. Vet J. 191(1):108-14 |
| PAX5 | Roche, Basel, Switzerland | Rabbit, IgG | SP34 | Ventana Benchmark XT | 1:200 | Canine indolent and aggressive lymphoma: clinical spectrum with histologic correlation. Aresu L, et al. Vet Comp Oncol. 2015 Dec;13(4):348-62. |
| MUM1 | Dako, Glostrup, Denmark | Mouse, IgG1 | MUM1p | Ventana Benchmark XT | 1:300 | Immunophenotypic Characterization of Canine Splenic Follicular-Derived B-Cell Lymphoma. Stein L. et al. Vet Pathol. 2019;56(3):350-357. |
| Ki-67 | Dako, Glostrup, Denmark | Mouse, IgG1 | MIB-1 | Tris-EDTA buffer, pH 9.0 | 1:50 | Immunophenotypic Characterization of Canine Splenic Follicular-Derived B-Cell Lymphoma. Stein L. et al. Vet Pathol. 2019;56(3):350-357. |
| Cleaved Caspase-3 | Cell Signaling Biotechnology, Leiden, The Netherlands | Rabbit, IgG | 5A1E | Citrate buffer, pH 6.0 | 1:200 | Canine Cutaneous Haemangiosarcoma: Biomarkers and Survival. Nóbrega DF, et al. J Comp Pathol. 2019; 166:87-96 |
| MYC | Santa Cruz Biotechnology, TX, USA | Mouse, IgG1 | 9E10 | Citrate buffer, pH 6.0 | 1:100 | Evaluation of NKX3.1 and C-MYC expression in canine prostatic cancer. Fonseca-Alves CE. et al. Res Vet Sci 2018; 118: 365-370 |

**Table S3.** Clinical results of 13 canine B-LL.

| **No.** | **Breed** | **Sex** | **Age** | **Weight (Kg)** | **Stage** | **Substage** | **PCV** | **PLT** | **LDH** | **Calcium** | **Extranodal involvement** |
| --- | --- | --- | --- | --- | --- | --- | --- | --- | --- | --- | --- |
| 1 | Mixed | m | 11 | 23.3 | V | a | normal | normal | normal | normal | no |
| 2 | Mixed | m | 12 | 9.1 | IV | a | normal | normal | increased | normal | no |
| 3 | Golden Retriever | fs | 8 | 30.8 | V | b | normal | normal | increased | normal | no |
| 4 | Dobermann | m | 7 | 37.5 | V | b | normal | normal | normal | normal | lung |
| 5 | Basset hound | fs | 10 | 24.9 | III | a | normal | normal | increased | normal | no |
| 6 | Golden Retriever | m | 7 | 33.1 | V | b | normal | normal | normal | normal | lung |
| 7 | Rottweiler | f | 5 | 41.8 | IV | a | normal | normal | normal | normal | no |
| 8 | Labrador | m | 8 | 32.1 | IV | b | decreased | decreased | normal | normal | no |
| 9 | Golden Retriever | fs | 10 | 33 | V | a | decreased | decreased | normal | normal | lung |
| 10 | Galgo | fs | 9 | 17.3 | V | a | normal | normal | normal | normal | no |
| 11 | Welsh Gorgi | fs | 5 | 15.6 | V | a | normal | normal | increased | normal | no |
| 12 | Beagle | m | 11 | 20.3 | IV | a | normal | normal | increased | normal | no |
| 13 | Mixed | mc | 9 | 31.8 | V | b | normal | decreased | increased | normal | no |

**Table S4.** Histologic variables of 13 canine B-LL.

| **No.** | **Mitotic count** | **Apoptotic Index (%)** |
| --- | --- | --- |
| 1 | 4.6 | 68.5 |
| 2 | 10.9 | 65 |
| 3 | 10.1 | 32.5 |
| 4 | 5.5 | 33.5 |
| 5 | 5.1 | 39.5 |
| 6 | 5.1 | 56 |
| 7 | 5.2 | 51.5 |
| 8 | 5.1 | 61.5 |
| 9 | 9.6 | 27 |
| 10 | 6.2 | 26 |
| 11 | 8.7 | 27.5 |
| 12 | 9.5 | 41 |
| 13 | 7.8 | 23.5 |
