# Supplementary figures and images for: Phenotypical Characterization and Clinical Outcome of Canine Burkitt-Like Lymphoma
Source: Front Vet Sci. 2021 Mar 17;8:647009. doi: 10.3389/fvets.2021.647009 (PMC8010238; doi:10.3389/fvets.2021.647009)

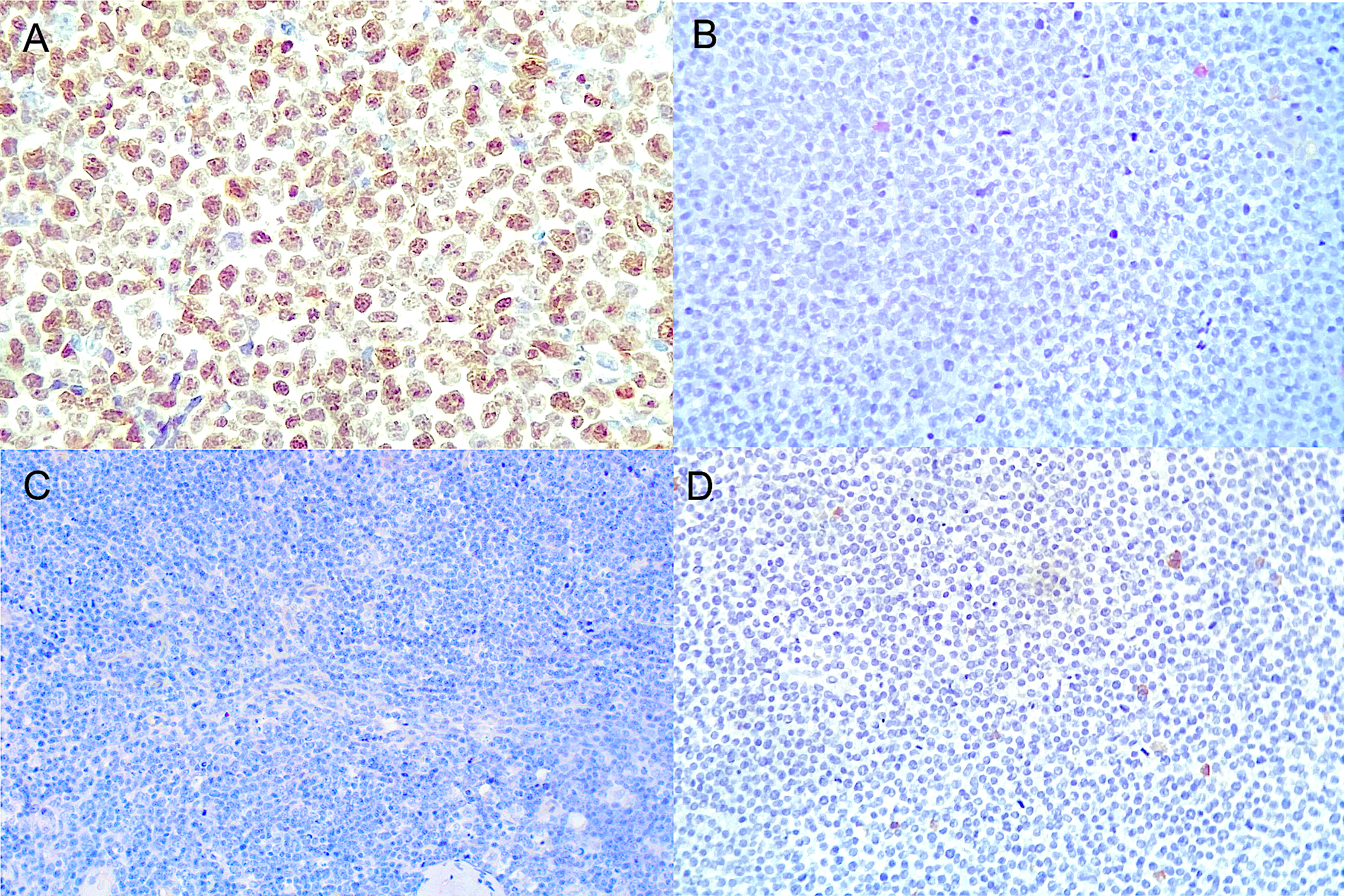

Supplement: Supplementary file 2 [file Image_1.TIF]
